# Functional Fiber Membranes with Antibacterial Properties for Face Masks

**DOI:** 10.1007/s42765-023-00291-7

**Published:** 2023-05-17

**Authors:** Papada Natsathaporn, Gordon Herwig, Stefanie Altenried, Qun Ren, René M. Rossi, Daniel Crespy, Fabian Itel

**Affiliations:** 1 Department of Materials Science and Engineering, School of Molecular Science and Engineering, Vidyasirimedhi Institute of Science and Technology (VISTEC), Rayong, 21210 Thailand; 2 Laboratory for Biomimetic Membranes and Textiles, Empa, Swiss Federal Laboratories for Materials Science and Technology, Lerchenfeldstrasse 5, 9014 St. Gallen, Switzerland; 3 Laboratory for Biointerfaces, Empa, Swiss Federal Laboratories for Materials Science and Technology, Lerchenfeldstrasse 5, 9014 St. Gallen, Switzerland

**Keywords:** Polydimethylsiloxane, Zinc oxide, Face mask, Electrospinning, Antibacterial

## Abstract

Reusable face masks are an important alternative for minimizing costs of disposable and surgical face masks during pandemics. Often complementary to washing, a prolonged lifetime of face masks relies on the incorporation of self-cleaning materials. The development of self-cleaning face mask materials requires the presence of a durable catalyst to deactivate contaminants and microbes after long-term use without reducing filtration efficiency. Herein, we generate self-cleaning fibers by functionalizing silicone-based (polydimethylsiloxane, PDMS) fibrous membranes with a photocatalyst. Coaxial electrospinning is performed to fabricate fibers with a non-crosslinked silicone core within a supporting shell scaffold, followed by thermal crosslinking and removal of the water-soluble shell. Photocatalytic zinc oxide nanoparticles (ZnO NPs) are immobilized on the PDMS fibers by colloid-electrospinning or post-functionalization procedures. The fibers functionalized with ZnO NPs can degrade a photo-sensitive dye and display antibacterial properties against Gram-positive and Gram-negative bacteria (*Escherichia coli* and *Staphylococcus aureus*) due to the generation of reactive oxygen species upon irradiation with UV light. Furthermore, a single layer of functionalized fibrous membrane shows an air permeability in the range of 80–180 L/m^2^s and 65% filtration efficiency against fine particulate matter with a diameter less than 1.0 µm (PM_1.0_).

**Graphical abstract:**

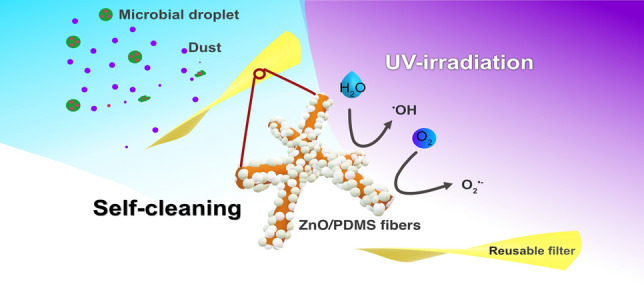

**Supplementary Information:**

The online version contains supplementary material available at 10.1007/s42765-023-00291-7.

## Introduction

After 3 years of the COVID-19 pandemic, an estimated 536 million people have been infected, about 6.3 million have died [1], and 10–20% of infected people have suffered from long-term side effects [2, 3]. Amid the spread of the virus, the global demand for face masks has reached approximately 449.5 billion in 15 months, including 129 billion face masks monthly at the beginning of the pandemic to reduce the transmission of the respiratory virus in public [4–6]. After use, face masks cannot be reused or recycled, hence worsening the microplastic problems [6, 7].

Textile-based face masks have been suggested as an alternative to disposable face masks because fabrics are available in large quantities and can be washed, even though they are prone to bacteria growth and accumulation of contaminants [8, 9]. However, friction and discharge during the laundry can induce a decrease in the filtration efficiency of woven and nonwoven filters [10–12].

Consequently, re-usable face masks could offer a solution to overcome the waste of clinical face masks while preventing bacteria growth and virus penetration, either without or complementary to conventional washing.

Several antimicrobial and self-cleaning materials were incorporated in or on fiber membranes. Commercial cellulose membranes were functionalized with disinfectants such as polyhexamethylene guanidine or neomycin sulfate to provide membranes with > 99% antibacterial and antiviral activities [13, 14]. Covalent grafting of the disinfectants on cellulose prevented their leaching during inhalation [15]. Besides biological antiviral agents [16, 17], which showed good inactivation properties against viruses, metal-based nanoparticles [18–23] display better stability [24], present low adhesion to microbes, and can denature specific proteins in microbes [25]. Functionalization of fabrics with Ag NPs [26–29], Au NPs [18, 30], and Cu NPs [31–34] provided effective antimicrobial properties against *Escherichia coli* (*E. coli*) or Herpes simplex virus type 1 (HSV-1). However, gold is expensive, and silver can be toxic [35]. Moreover, Cu NPs can increase the potential risk of skin burns under solar irradiation [34]. Carbon-based materials [36, 37] were also used to prevent the adhesion of microbes on face masks. Heat generation of up to 80 °C, however, required sunlight and can potentially reduce material’s strength for long-term applications [38].

Metal oxide nanomaterials such as TiO_2_ [39–41], ZnO [39, 42, 43], and CuO [39, 44] are photoactive and used for self-cleaning or self-sterilization purposes due to their ability to catalyze the generation of electrons on their surface, especially upon photoirradiation. In the presence of oxygen from the air, the electrons generate reactive oxygen species (ROS), including superoxide anion radicals (^·^O_2_^–^), hydrogen peroxide (H_2_O_2_), singlet oxygen (^1^O_2_), and hydroxyl radicals (^·^OH). ROS are known to kill bacteria and viruses by destroying their membranes or capsid proteins [45, 46]. Additionally, these metal oxide NPs can produce ROS for a long time. For example, free-standing TiO_2_ nanowires could be reused more than 1000 times [47]. For antibacterial applications, ZnO displayed a higher toxicity to *B. subtilis* than TiO_2_ nanoparticles [48] and to *E. coli* than both TiO_2_ and CuO [39].

Functionalizing face masks or fabrics with metal oxide nanomaterials by deposition [49–52], grafting [20], and electrospinning [53, 54] have produced materials with outstanding self-cleaning and antimicrobial properties. Polypropylene (PP) is the main filtering material for nonwoven textiles, such as filtration membranes and face masks [55]. PP has many advantages such as low density, good chemical stability, low melting temperature and hydrophobicity that are suitable for fabricating nonwoven microfibers (diameter of ~ 1–10 µm) via spun bond and melt-blowing processes [56], but degrades under UV irradiation [57, 58]. Therefore, functionalizing PP nonwoven materials with photocatalysts can limit their durability, which is an issue for reusable self-cleaning membranes.

Polydimethylsiloxane (PDMS) and polytetrafluoroethylene (PTFE) are hydrophobic polymers with high stability against photo-, thermal- and chemical degradation. PDMS [59, 60] and PTFE [61, 62] have been blended with metal oxide nanomaterials to provide reusable superhydrophobic self-cleaning surfaces. In a rather complex procedure, a PTFE filtration membrane was treated with nitrogen plasma to generate radicals for grafting a hydrophilic polymer. The metal precursor was assembled on the grafted PTFE by coordination between metal and hydrophilic polymer and was oxidized to form metal oxides. On the other hand, metal oxides can be conveniently grafted on hydrophobic PDMS with covalent linkages by simple UV treatment [63, 64]. The covalent bonding between metal oxide nanoparticles and silanol groups can possibly prevent particle detachment and the inhalation of particles. PTFE can be fabricated as a fibrous membrane with cold-pressing [65]. However, uncured PDMS is difficult to be processed as fibers via classical techniques due to the very low glass transition temperature of PDMS (− 150 °C) [66]. Despite this disadvantage, PDMS fibers were successfully generated by the electrospinning technique using a spinnable polymer shell scaffold around an uncured PDMS core. Electrospinning generates nonwoven membranes of nanofibers by applying a high electric potential to a polymer solution. Due to the small fiber diameter, these membranes possess extremely high surface-to-volume ratios and high porosity, which are both properties that are highly attractive for filtration applications. Electrospinning of core–shell PDMS/polyvinylpyrrolidone (PVP) followed by in-situ curing of the PDMS core has been reported [67, 68] without further functionalization for self-cleaning and antimicrobial properties.

The fabrication of photocatalyst/PDMS composite fibers by electrospinning and their photocatalytic activity have been reported [69, 70]. However, non-covalent bonding between the photocatalysts and PDMS could lead to the detachment of the photocatalyst after use. Recently, PDMS was grafted on photocatalyst nanoparticles dispersed in an organic solvent by covalent bonding via a simple UV treatment [63]. However, there is no report on the functionalization of PDMS fibers with photocatalyst by using UV irradiation to functionalise.

**Fig. 1:**
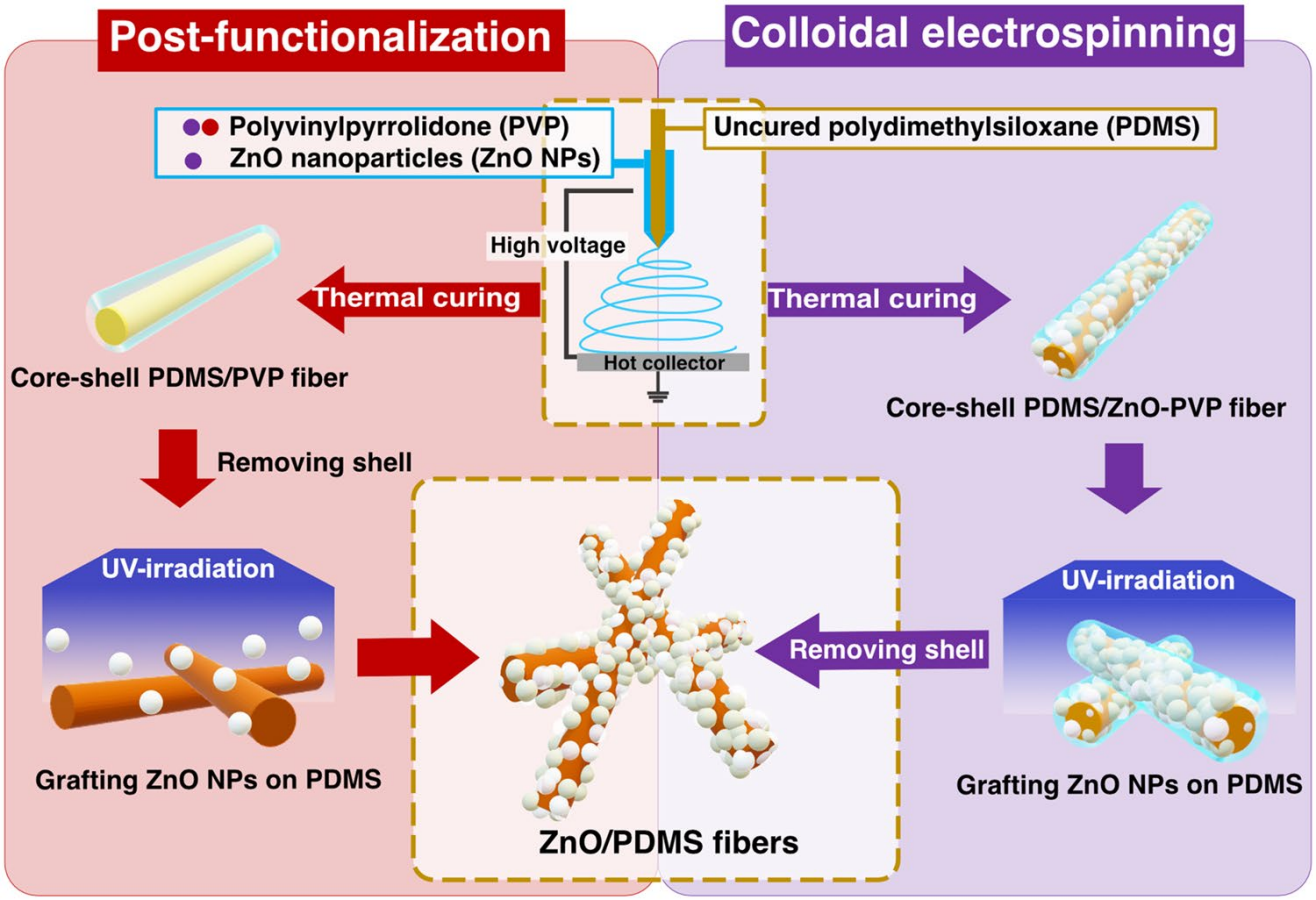
Scheme depicting the fabrication of ZnO/PDMS fibers by two different methods: post-functionalization of electrospun fibers (left side) and colloidal electrospinning. In the post-functionalization (PO) process, core–shell PDMS/PVP fibers without ZnO NPs are electrospun, and ZnO NPs are then grafted after PVP-shell removal on PDMS fibers via UV irradiation. In the colloidal electrospinning (CE) process, PDMS and colloidal ZnO-PVP dispersion are electrospun, and the ZnO NPs were grafted on PDMS with UV irradiation followed by removal of PVP

Herein, we report the development of a PDMS-based antibacterial fiber membrane that can be used for face masks. We functionalize ultrafine PDMS fibrous membranes with photocatalyst by UV irradiation and investigate their photocatalytic activity, antibacterial activity, and filtration efficiency. The membrane can deactivate bacteria when irradiated with UVA light and, thus, can be reused. Here, we selected ZnO NPs, as they generate ROS both under UV–VIS irradiation and in the dark [71]. PDMS fibers were prepared by coaxial electrospinning with in-situ thermal curing and subsequent functionalization with ZnO NPs via post-functionalization (PO) or colloidal electrospinning (CE) under UV irradiation (Fig. 1).

## Experimental Section

### Materials

Polyvinylpyrrolidone (PVP, Mw ~ 1,300,000 Da, Sigma Aldrich), Sylgard 184 silicone elastomer (Dow Europe), Sylgard 184 curing agent (Dow Europe), zinc oxide nanopowders (ZnO NPs, < 50 nm, > 97%, Aldrich), rhodamine B (Microscopy, Merck), methanol (MeOH, ≥ 99.9%, analytical reagent grade, Fisher Scientific), 2-propanol (IPA, RPE grade, Carlo Erba) and ethanol (EtOH, 99.9%, QRëC) were used as received. Demineralized water was used throughout the study. A 304 stainless steel mesh (wire diameter of 0.061 mm and pore width of 0.108 mm, Paphavin product), plate-count-agar (agar 12 g/L, Sigma Aldrich) and a polylactide (PLA) mesh (Sichuan KST Automatic Equipment, China) were used without any pre-treatment.

### Electrospinning of PDMS/PVP Core–Shell Fibers

The fabrication was developed based on a previous report [68]. The PDMS core solution consisted of 0.4 g of Sylgard 184 elastomer and 0.04 g of curing agent (9:1 w/w ratio) that was freshly prepared just before electrospinning. For the shell solution, 1.6 g of PVP was dissolved in 8.4 g MeOH to make a 16 wt% PVP solution. The solutions were filled into separate 1 mL syringes (inner diameter of 4.78 mm) and mounted onto syringe pumps (Fusion 100, Chemyx). The solutions were pumped at flow rates of 0.1 mL/h for the inner and 0.4 mL/h for the outer channels of a blunt-ended coaxial spinneret (Ramé-Hart instrument). The diameters of the inner and outer channels were 0.406 mm (22G) and 0.840 mm (18G), respectively. The electrospinning (TL-Pro-BM, Tong Li Tech) was performed for 2 h with a positive high voltage of 11 kV at the needle, a needle-to-collector distance of 15 cm, a grounded heating plate covered with aluminum foil, and a set temperature of 100 °C, a relative humidity between 40 and 50% and a room temperature between 25 and 35 °C. After spinning, the support material (aluminum foil or wire mesh) containing the fibrous PDMS/PVP membranes was removed from the hot collector and further cured in an oven for 24 h at 100 °C to crosslink the PDMS core.

### Post-functionalization of PDMS Fibers with ZnO

The electrospun PDMS/PVP core–shell fibers were carefully peeled from the aluminum foil and placed on the PLA mesh (5 × 7 cm). PVP on PDMS/PVP fibers was removed by washing 3 times with 5 mL of EtOH and then carefully drying with a stream of air. The PDMS fiber membranes on PLA mesh were then cut into squares (2 × 2 cm) and placed in a polypropylene box with 3 × 3 cm wells. 1.68 mL of ZnO NPs in IPA (0.108, 0.168 and 0.350 mg/mL) was pipetted into the wells containing the PDMS membranes and exposed to UV irradiation (UVA, λ = 365 nm, 5 mW/cm^2^) using a UVA LED lamp (Awellcure, China) for 2 h. The irradiated fibers were washed with 2 mL of EtOH and dried with air to obtain fibrous ZnO/PDMS membranes prepared with three different ZnO NP concentrations of 4.3 (PO_low), 6.7 (PO_medium), and 14.1 (PO_high) wt% ZnO.

For pressure drop and filtration testing, the PDMS/PVP fibers were electrospun on round stainless steel meshes (diameter of 6 cm). PVP was removed by washing three times with 5 mL of EtOH. The samples were placed in crystal bathes (diameter of 7.6 cm) containing 9.1 mL of 0.108, 0.168 or 0.35 mg/mL ZnO NPs dispersion in IPA. The fibers were irradiated with UVA (5 mW/cm^2^) for 2 h. Then, the ZnO/PDMS membranes were washed with 5 mL EtOH to remove unbound ZnO NPs and air-dried.

### Colloid Electrospinning (CE) of ZnO/PVP and PDMS

ZnO NPs (19.0, 37.8 or 62.7 mg) were dispersed in 0.72 g of MeOH and sonicated for 5 min. The ZnO dispersions were then transferred to vials containing the solution of PVP in MeOH to obtain a final PVP concentration of 16 wt% and mixed mechanically using a spatula for 1 min.

Electrospinning and curing were performed according to the conditions described before. The thermally cured PDMS/ZnO-PVP membranes with three different ZnO:PVP weight ratios of 0.05:0.95 (CE_low), 0.10:0.90 (CE_medium) and 0.16:0.84 (CE_high) were irradiated with UVA (5 mW/cm^2^) for 2 h to graft ZnO NPs on the PDMS [63]. For the antibacterial testing, the CE fiber membranes were removed from the aluminum foil and placed on PLA meshes with a width of 5 cm and a length of 7 cm and rinsed 3 times with 3 mL of EtOH. For the filtration efficiency tests, the fibers were electrospun on stainless steel meshes. PVP was removed by carefully rinsing the membranes 3 times with 5 mL of EtOH and carefully drying them under a stream of air.

### Photocatalytic Activity of ZnO/PDMS Membrane

3 mg of ZnO/PDMS membranes were placed in borosilicate test tubes containing 5 mL of 10.4 µM rhodamine B in water. All test tubes were kept in the dark for 30 min and exposed to UV irradiation (5 mW/cm^2^). The rhodamine B solutions were agitated during the reaction by magnetic stirring. 100 µL of rhodamine B in the presence or absence of fiber membranes were taken from the tubes and placed in a 96-well plate at different time points of accumulated UV irradiation time for measuring light absorbance at 555 nm. The concentration of rhodamine B versus time was fit with a pseudo-first-order equation in Eq. (1):

1 $$\ln \left( {\frac{{C_{0} }}{C}} \right) = kt,$$

where *C*_0_ is the initial concentration of rhodamine B, *C* is detected concentration, *k* is the rate constant (min^−1^) and *t* is time (min).

### Antibacterial Activity of ZnO/PDMS Membrane

For the preparation of the bacterial solutions, a single colony of *E. coli* DSMZ 1103 and *Staphylococcus aureus* ATCC 6538 were picked from a Plate-Count Agar (PC-Agar, Sigma Aldrich 70152) plate and added to 5 mL of a solution containing 30 wt% Tryptic Soy Broth (TSB, Sigma Aldrich 22092) and 0.25 wt% glucose. The suspensions were incubated at 37 °C with shaking at 160 rpm overnight. The bacterial culture was diluted with 1 × phosphate-buffered saline (PBS, Sigma P4417) to 0.1 optical density at 600 nm (OD600 nm). The suspensions were further grown for 1.5 h to obtain exponentially growing cells. The *E. coli* and *S. aureus* suspensions were diluted again to 2.5 × 10^4^ colony-forming units (CFU)/mL and 6.14 × 10^5^ CFU/mL, respectively. The PDMS and ZnO/PDMS fiber membranes were punched to form circles (with a diameter of 8 mm) with a stainless punch and placed in a 48-well plate. 25 µL of the diluted bacteria suspensions were loaded onto the punched fibers and incubated for 2 h at room temperature. The fiber membranes were treated with UV irradiation (365 nm, 5 mW/cm^2^) for 15 min while the plates were cooled on ice, using untreated samples as controls. 20 µL of the *E. coli* and *S. aureus* suspension was taken from the fiber membrane surface and loaded on agar plates after series dilutions for colony counting. This is for the measurement of the planktonic cells in the suspension. The adherent cells on the fiber membranes were analyzed by placing the fiber samples on the bacteria spread on agar plates for 5 min. The plates were incubated at 37 °C for 18 h. The number of colonies of bacteria on agar plates was counted by an automatic colony counter (Scan 300, Interscience). The total viable cells were calculated as the planktonic plus the adherent cells.

### Particle Filtration and Air Permeability Analysis

An aerosol generator (AGK2000, PALAS), a corona discharge neutralizer (CD2000, PALAS), a particle analyzer (DMS5000, Cambustion), a sample holder and pumps were installed according to a previous report (see Fig. S1) [72]. The airflow velocity was fixed at 8 cm/s. Aerosols were generated from a fructose solution (20 g/L) to obtain a final aerosol concentration of 3.5 mg/m^3^. The aerosol was fabricated from a nebulized fructose solution instead of a NaCl solution used in some studies [73], as this ensured the production of sufficient amounts of particles over a wider range of sizes simultaneously in one experiment, and hence facilitated classification according to a higher number of potentially applicable norms and references. The filtration efficiency (FE) was calculated from the measured concentrations of particles before (*C*_0_) and after (*C*) filtration with Eq. (2):

2 $${\text{FE}} = 1 - \frac{C}{{C_{0} }}.$$

The air permeability was determined using an accredited self-built flowmeter.

### Analytical Tools

The morphology of electrospun fibers was observed with a scanning electron microscope (SEM, S-4800, Hitachi High-Technologies). The fiber samples were placed on carbon tape and were sputter-coated (high vacuum coater EM ACE600, Leica) with a 7 nm layer of gold/palladium. The internal fiber structure was monitored with a transmission electron microscope (TEM, JEOL-ARM200F, JEOL). For this, the fibers were directly electrospun on copper grids (standard square 300 mesh, G300CU, Electron Microscopic Science). Elemental analysis of the ZnO immobilized PDMS fibers was performed using energy-dispersive x-ray spectroscopy (EDX, X-max 150 mm^2^ silicon drift detector) coupled with the SEM (JSM-7610F, JEOL) apparatus. TGA thermograms of the fiber membranes in alumina crucibles were recorded with a thermogravimetric analyzer (NETZSCH TG209 F1 Iris instrument, NETZSCH-Gerätebau GmbH) with a nitrogen flow rate of 50 mL/min and a heating rate of 10 K/min. The mechanical properties of PDMS and ZnO/PDMS fibrous membranes were measured with a tensile tester (eXpert 5604, ADMET) at a strain rate of 10 mm/min and a grip separation length of 2 cm. The fibrous membranes were cut with a rectangular cutting die (D-1708 MOD, PIONEER Die-tecs) with a dimension of 0.5 × 3.5 cm. The thicknesses of the membranes were measured with a dial thickness gauge (aml instruments). The inhalation through the membranes was simulated by covering one end of a rubber tube (diameter 5 mm) with PO_high or CE_low membranes. The other end of the tube was connected to a vacuum pump (V100, Buchi). A vacuum was applied through the membrane with a hysteresis of 100 mbar (800–900 mbar) for 15 min. The absorbance of rhodamine B was detected at 555 nm with a microplate reader (Infinity 200 pro, Tecan, 9 nm bandwidth) equipped with a Xenon light source and a silicon photodiode detector.

### Data Reporting and Statistical Analysis

Each antibacterial test was performed in three replicates per fiber membranes (PO_low, PO_medium, PO_high, CE_low, CE_medium, CE_high and PDMS). Colony forming units per area of membrane (CFU/cm^2^) was calculated and then plotted on a logarithmic scale (log CFU/cm^2^). Statistical significance was defined with a 95% confidential interval (*P* < 0.05) calculated by a one-way ANOVA analysis with a Tukey mean comparison.

## Results and Discussion

### Preparation and Characterization of Control PDMS/PVP Core–Shell Fibers

**Fig. 2:**
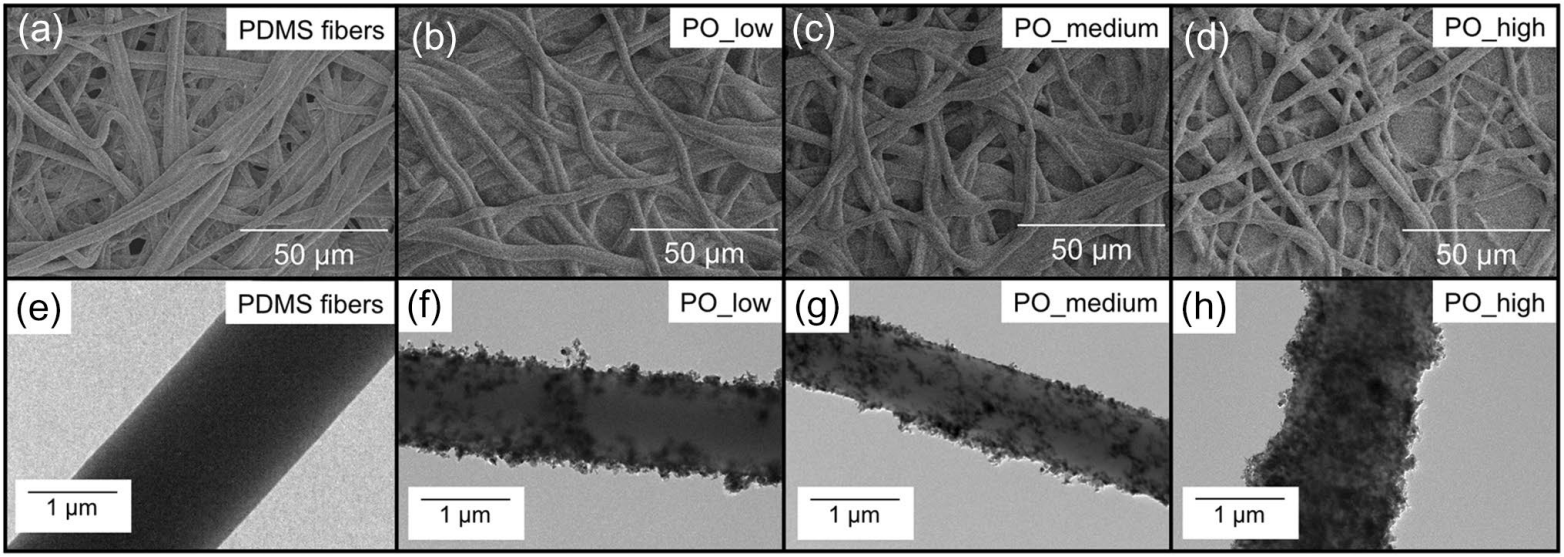
SEM (**a**–**d**) and TEM (**e**–**h**) micrographs of PDMS fibers (**a**, **e**) and ZnO/PDMS fibers prepared by post-functionalization containing (**b**, **f**) 2.7 wt% ZnO (PO_low), (**c**, **g**) 5.6 wt% ZnO (PO_medium), and (**d**, **h**) 6.9 wt% ZnO (PO_high)

Uncured polydimethylsiloxane (PDMS) was successfully electrospun with poly(vinyl alcohol) (PVA) or poly(vinyl pyrrolidone) (PVP) using a coaxial needle. PVA and PVP were selected as polymeric shells because they are immiscible with PDMS and have a high solubility in polar protic solvents, such as water for PVA and PVP, and alcohols for PVP. With the PVA shell, the PDMS/PVA fibers exhibited a flat-ribbon shape (Fig. S2a) due to the low glass transition temperature (*T*_g_) of PVA (76 °C) [74], which is lower than the curing temperature of 100 °C used here for PDMS. On the other hand, PVP has a higher glass transition temperature (*T*_g_ = 172 °C) [75] above the curing temperature of PDMS on the hot collector, and the PDMS core was kept in shape as round fibers and could be further cured for another 24 h at 100 °C until complete cross-linking of PDMS (Fig. S2b). SEM and TEM images show that PDMS is surrounded by a shell of PVP. The generated fibers are homogenous without beads and show an average diameter of 2.05 ± 0.24 μm. The presence of PVP and PDMS was further confirmed by thermogravimetric analysis (TGA) as shown in Fig. S2c. The calculated PVP:PDMS weight ratio from TGA is around 30:70 matching the feed PVP:PDMS weight ratio of 34:66, indicating there was no spill of PDMS or PVP during electrospinning. After the removal of the PVP shell from the cured PDMS/PVP fiber membranes with MeOH, the average diameter of PDMS fibers was 1.52 ± 0.21 µm (Fig. 2a, e). The nitrogen content decreased from 11.4 at.% for PDMD/PVP fibers to 0.1 at.% for PDMS fibers, as detected by EDX spectroscopy (Table 1), hence, indicating an efficient removal of the PVP shell. Moreover, the carbon-to silicon-atomic ratio decreased from 7:1 (PDMS/PVP) to 2.5:1 (PDMS fibers after PVP removal).

**Table 1:** Surface elemental analysis of PDMS-based fibers using energy-dispersive X-ray spectroscopy (EDX) on the surface of the fibers

Entry	Content of element (at.%)					C:Si atomic ratio	Content of ZnO (wt%)	
	C	N	O	Si	Zn		Theoretical	Experimental
PDMS/PVP fibers	59.4	11.4	20.6	8.5	0.1	7:1	–	–
PDMS fibers	52.0	0.1	26.8	21.1	0.2	2.5:1	–	–
PO_low	50.5	0.1	32.1	16.7	0.7	3:1	4.3	2.7 ± 1.9
PO_medium	49.2	0.2	30.5	18.8	1.4	2.6:1	6.7	5.6 ± 1.4
PO_high	48.8	3.3	24.5	21.9	1.6	2.2:1	14.1	6.9 ± 3.3
CE_low	50.5	0.3	27.4	21.6	0.2	2.3:1	2.1	1.4 ± 0.5
CE_medium	50.1	0.1	30.0	19.7	0.2	2.5:1	4.3	1.2 ± 0.6
CE_high	49.3	0.3	27.0	23.1	0.3	2.1:1	7.1	1.4 ± 0.6

Comparison between theoretical and experimental content of ZnO in PDMS fibers

### Preparation and Characterization of PDMS Fibers with ZnO

To equip the fibers with antimicrobial properties, the PDMS fibers were functionalized with ZnO nanoparticles (ZnO NPs) by two different methods: post-functionalization (PO) of fibers and colloidal electrospinning (CE) by using low, medium and high concentrations of ZnO NPs for both methods.

By the post-functionalization method, the ZnO NPs are well distributed and immobilized on PDMS as shown in PO_low, PO_medium, and PO_high with theoretical ZnO contents of 4.3, 6.7 and 14.1 wt%, respectively (Fig. 2). The actual ZnO content on PDMS increased by increasing the ZnO loading concentration; on the other hand, the experimentally determined ZnO content by energy-dispersive X-ray spectroscopy (EDX) was lower, 2.7 ± 1.9, 5.6 ± 1.4 and 6.9 ± 3.3 wt% (Table 1 and Fig. S4). Due to the fact that the functionalization is an interfacial reaction, only ZnO NPs close to the PDMS surface can be grafted on PDMS, and unreacted ZnO NPs in dispersion were then removed. Lower and higher concentrations of ZnO were also investigated (Fig. S5). When the loading concentrations of ZnO on PDMS were 1.4 and 2.2 wt%, the ZnO content on the PDMS fibers was lower than the detection limit of EDX. When the ZnO loading was increased to 22, 43 and 67 wt%, ZnO aggregates formed. This is due to the avoidance of stabilizers or adsorbed organic molecules. Photoactive nanoparticles tend to aggregate under UV irradiation because of the formation of a hydroxyl bridge between photoactive nanoparticles [76, 77].

**Fig. 3:**
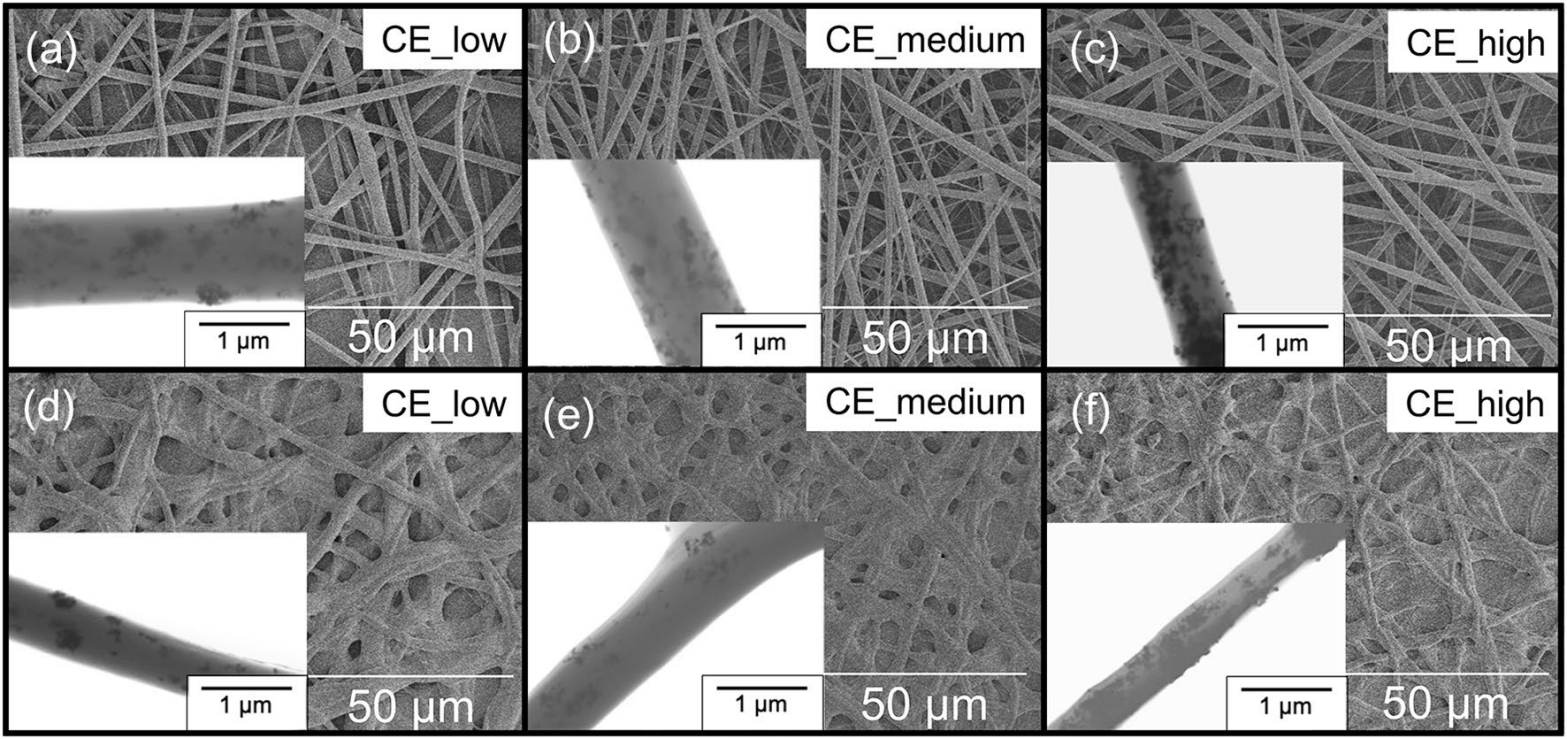
SEM and TEM (insets) micrographs of ZnO/PDMS fibers prepared by colloidal electrospinning before (**a**–**c**) and after (**d**–**f**) PVP removal containing **a**, **d** 2.1 wt% ZnO (CE_low), **b**, **e** 4.3 wt% ZnO (CE_medium) and **c**, **f** 7.1 wt% ZnO (CE_high)

By the colloidal electrospinning method, different concentrations of ZnO were added to the PVP solution and the dispersions were electrospun using a coaxial needle (Fig. 1). After UV irradiation, smooth PDMS/ZnO-PVP fibers without beads and leaking of PDMS were obtained as shown in the SEM micrographs (Fig. 3a–c). Increasing the actual ZnO content did not significantly change the average fiber diameter ranging from 2.11 ± 0.58, 2.26 ± 0.48 and 2.30 ± 0.41 µm for CE_medium, CE_low and CE_high, respectively. Moreover, a high concentration of ZnO on PVP shell was observed in CE_high compared to CE_low and CE_medium as shown in TEM micrographs (Fig. 3a–c). At even higher ZnO concentrations (10.7 wt%), the average diameter decreased to 1.40 ± 0.71 μm (Fig. S6) compared to CE_low, CE_medium and CE_high, which is most probably due to the precipitation of ZnO during electrospinning. A higher ZnO content in the PVP solution increases the hydrophilicity of the ZnO-PVP dispersion and hence increased interfacial tension between PDMS and the dispersion. A large interfacial tension can induce instability of the Taylor cone and the Rayleigh–Plateau instability triggering the break-up of the core [78].

After PVP shell removal, the morphology of ZnO NPs on PDMS fibers was investigated by SEM and TEM (Fig. 3d–f). TEM images showed a strong decrease of ZnO NPs concentration on PDMS fibers (Fig. 3d–f), which was confirmed by EDX analysis with concentrations lying between 1.2 and 1.4 wt% (Table 1). In contrast to the post-functionalization method, the ZnO content did not increase with increasing initial ZnO concentration in the PVP/ZnO dispersion (Fig. S4) and remained low for all samples. Thus, the ZnO content could not be controlled by colloidal electrospinning and was rather arbitrary. Apparently, the majority of ZnO NPs on fibers prepared by colloidal electrospinning were removed during PVP removal, indicating that only ZnO NPs in close contact with the PDMS surface were grafted on.

Thicknesses of PDMS/PVP and CE_low fibrous membranes before shell removal were 106 ± 7 µm and 64 ± 19 µm, respectively, as detected by SEM (Fig. S7a, b). After functionalization and shell removal, the thicknesses of PO_high and CE_low were 24 ± 4 and 19 ± 7 µm, respectively (Fig. S7c, d). The tensile strengths of PDMS, PO_high and CE_low were 1.7 ± 0.3, 0.7 ± 0.2 and 0.8 ± 0.3 MPa, respectively (Table S1). The tensile strengths of ZnO/PDMS fibrous membranes were 34 times higher than electrospun TiO_2_-PDMS composite fibrous membranes [69]. PDMS/PVP and PDMS/ZnO-PVP fibrous membranes before functionalization could be peeled easily from the stainless mesh (Fig. S8a, b). After functionalization and shell removal, the PO_high was opaque while CE_low was translucent (Fig. S8c, d).

A vacuum pressure of 100 mbar was applied through the ZnO/PDMS membranes (PO_high and CE_low) to simulate inhalation through the membrane. This applied pressure was 25 times higher than the reported human inhale pressure (4 mbar) [79]. Then, ZnO content on PDMS was measured by EDX spectroscopy. ZnO contents in PO_high and CE_low did not significantly decrease by applying the vacuum (Fig. S9), indicating that the composite fibers were stable.

### Photocatalytic Activity of ZnO/PDMS Membrane

**Fig. 4:**
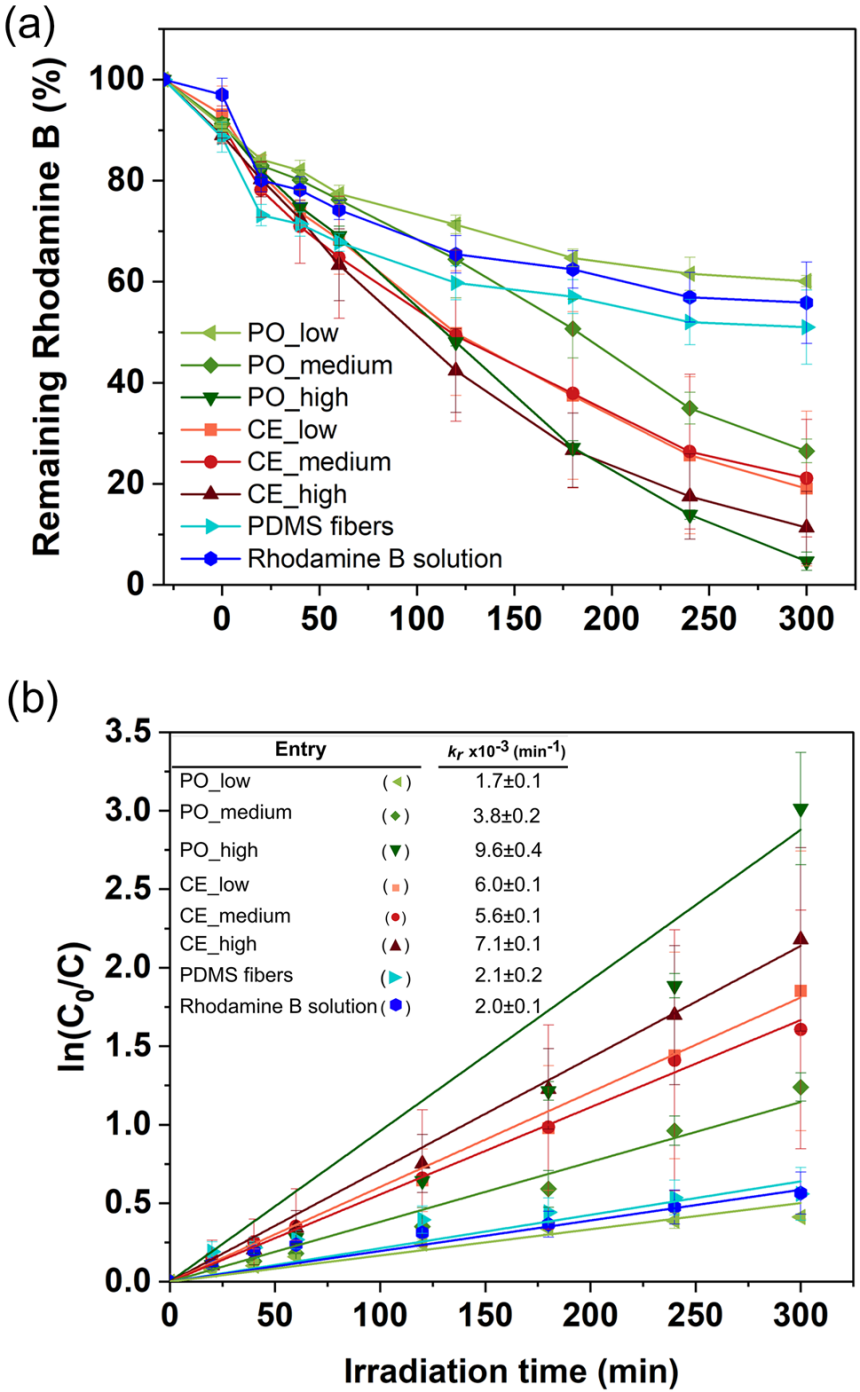
Photodegradation profile (**a**) and kinetics and rate constants (*k*_r_) (**b**) of rhodamine B photodegradation under UV irradiation with ZnO/PDMS fibers produced by post-functionalization (PO) or colloidal electrospinning (CE)

The photocatalytic performance of ZnO on the PDMS fibers was assessed by monitoring the degradation of rhodamine B, a model dye typically investigated for photodegradation studies in water [80–83]. A solution of rhodamine B without fiber membranes was irradiated with UV for 5 h, and the concentration of rhodamine B decreased to 56% of its initial concentration (Fig. 4a). The concentration rhodamine B in presence of ZnO/PDMS fibers prepared by the PO method (PO_medium and PO_high) decreased by 55 and 91%, respectively, after 300 min of UV irradiation but the photodegradation of rhodamine B could not be catalyzed with PO_low (Fig. 4a). The photodegradation of rhodamine B with fibers prepared from CE was similar to PO_medium and PO_high even though the ZnO content of fibers prepared by CE were lower. The TEM images of fibers prepared by PO (Fig. 2d–f) and CE (Fig. 3d–f) indicate, that the CE method possibly provides a lower shielding effect of UV by ZnO than the PO because the ZnO NPs are more homogeneously distributed on the fiber surface allowing better penetration of UV through the PDMS fiber to activate ZnO on the opposite side of the fiber. Therefore, fibers prepared by CE had higher efficiency than fibers prepared by PO to generate ROS to deplete rhodamine B.

The kinetics of degradation of ZnO/PDMS fibers could be fitted with a pseudo-first-order kinetic Eq. (1) (Fig. 4b). The kinetic rates increased with increasing ZnO content of ZnO/PDMS fibers prepared by PO. The degradation rate of rhodamine B using PO_low (*k*_*r*_ = (1.7 ± 0.1)⋅10^–3^ min^−1^) was similar to control PDMS membranes without ZnO because the ZnO content of PO_low was low. On the contrary, even though the ZnO content of CE fibers was lower than PO_low and PO_medium, kinetic rates of rhodamine B degradation using fibers prepared by CE were similar ranging from (6.0 ± 0.1)⋅10^–3^, (5.6 ± 0.1)⋅10^–3^ and (7.1 ± 0.1)⋅10^–3^ min^−1^ for CE_low, CE_medium and CE_high and were higher than PO_medium (*k*_r_ = (3.8 ± 0.2)⋅10^–3^ min^−1^).

To show that the membranes can be reused multiple times, droplets of rhodamine B solution were applied on the membranes, illuminated with UV light for 1 h and the degradation was observed by the decoloration of the rhodamine B solution (Fig.  S10). After washing the surface with EtOH, the whole procedure was repeated five times. On PDMS membranes without ZnO NPs, the solution remained red, showing that UV alone cannot degrade the dye. In the case of PO_high, a complete decolorization was observed for five cycles. CE_low, on the other hand, was only able to effectively photodegrade the dye for two cycles. This decrease in photocatalytic activity can be attributed to the dissolution of zinc over time [84]. As CE_low showed a lower zinc content than PO_high, it is expected that PO_high also loses its photocatalytic activity over time. (Fig. S10).

### Antibacterial Activity of ZnO/PDMS Membrane

**Fig. 5:**
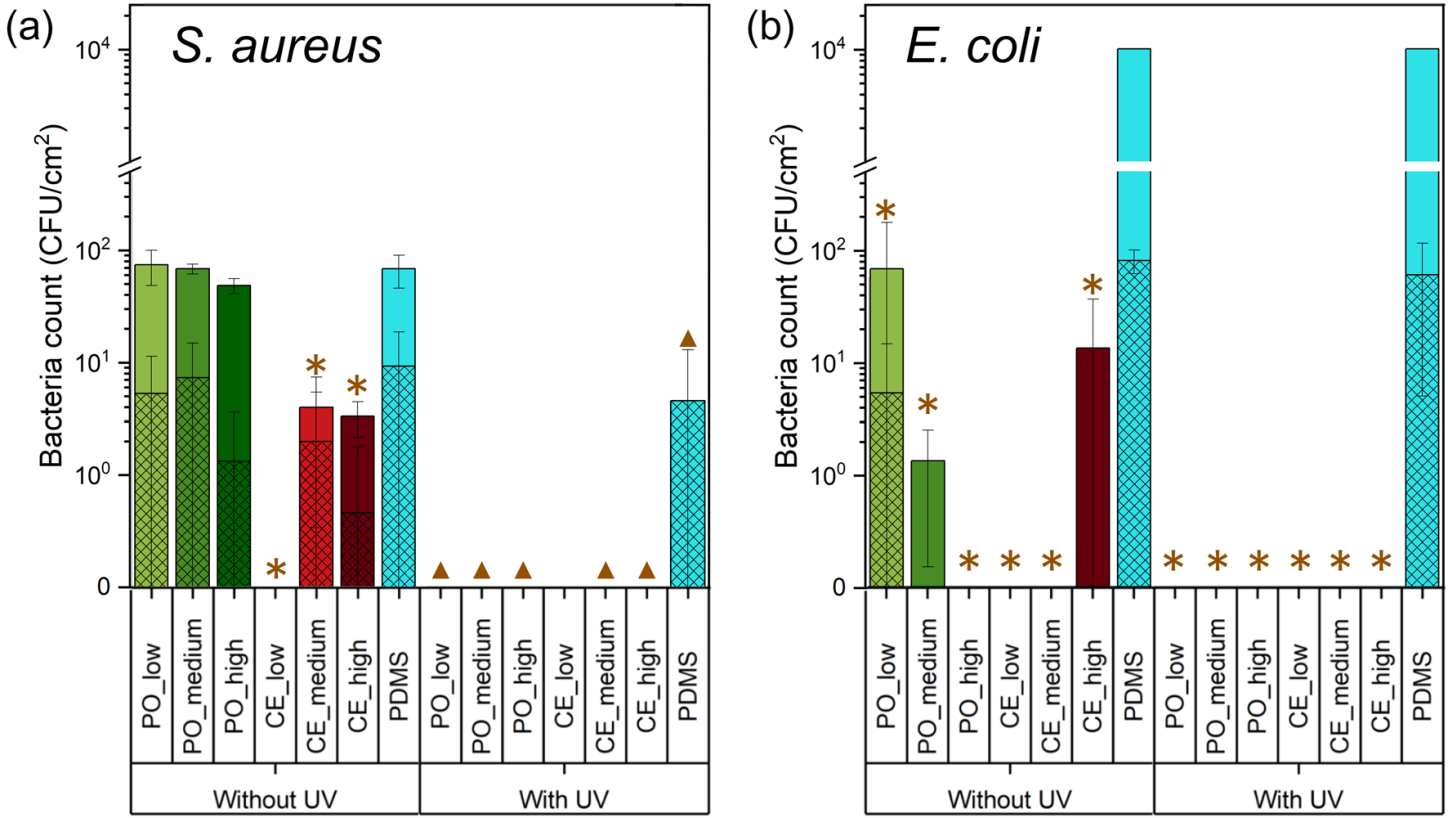
Antibacterial activity (average bacteria count, CFU/cm^2^) of PDMS control fibers and ZnO/PDMS fibers prepared by colloidal electrospinning (CE) and post-functionalization (PO) against Gram-positive *Staphylococcus aureus* (*S. aureus*) (**a**) and Gram-negative *Escherichia coli* (*E. coli*) (**b**) using contact test (grid pattern) and spotting test (no pattern). Significance was determined using a single *t* test of the log reduction data points with *P* ≤ 0.001. *Significantly different from PDMS. Filled triangle significantly different from without UV

Bacterial adhesion is an important aspect to assess the antibacterial effect of ZnO/PDMS fiber membranes. Here, antibacterial tests were conducted using Gram-negative *E. coli* and Gram-positive *S. aureus*. A total volume of 25 µL of the bacterial suspension was added to the sample discs yielding a theoretical starting colony of 1250 CFU/cm^2^ for *S. aureus* (Fig. 5a) and 30,700 CFU/cm^2^ for *E. coli* (Fig. 5b). Bacteria were then incubated for 2 h before starting the bacterial counting analysis. Without UV irradiation, the number of viable *S. aureus* remained at similar CFU levels for all PO samples compared to the PDMS control (68 ± 25 CFU/cm^2^). In contrast to the PO samples, the CE samples showed a significant reduction of viable cells of 100% for CE_low, 94% for CE_medium (4 ± 7 CFU/cm^2^) and 96% for CE_high (3 ± 2 CFU/cm^2^). With UV irradiation, there was a 100% reduction in viable *S. aureus* on all tested samples with ZnO-functionalized fibers irrespective of the ZnO concentration and the fiber material, whereas the control PDMS still showed some detectable colonies with 4 ± 7 CFU/cm^2^ (Fig. 5a).

For *E. coli*, all samples with ZnO-functionalized fibers showed a significant reduction of viable bacteria, irrespective of whether UV irradiation was used, compared to the PDMS control, whereas the number of bacteria on the control samples even increased above the starting concentration of 3.0 × 10^4^ CFU/cm^2^ (Fig. 5b). The viability of *E. coli* on CE_low without UV irradiation was 0 CFU/cm^2^ while it was 13 ± 23 CFU/cm^2^ for CE_high. Therefore, the antibacterial activity of CE_low and CE_high was statistically not different (*P* > 0.05), probably due to the similar actual concentration of ZnO in CE_low (1.4 ± 0.5 wt%) and CE_high (1.4 ± 0.6 wt%). As shown by the large number of CFU in the supernatant, *E. coli* proliferated much better on the PDMS control samples than *S. aureus*. It has to be noted, that even though the initial starting colony concentration of *E. coli* was 10-times higher than for *S. aureus*, the number of adhered bacteria (grid patterned bars in Fig. 5) was for both bacteria 10-times below the starting concentration, showing that both bacteria adhered the same on the PDMS surface. Regarding the photoactivation, the samples with ZnO-functionalized fibers strongly enhanced the bactericidal activity towards *S. aureus,* while the samples themselves possess high bactericidal activity towards *E. coli* even without the aid of photo activation, demonstrating that *S. aureus* and *E. coli* have different sensitivity towards the ZnO samples, similar to what we reported previously [85]. Due to their thicker bacterial cell wall, Gram-positive bacteria (here *S. aureus*) are more resistant to ROS than Gram-negative (here *E. coli*). The inactivation of *E. coli* cells by ZnO without UV irradiation might be attributed to the fact that ZnO NPs can generate small concentrations of ROS even in the dark, and, thus, could harm or penetrate the thin bacterial cell wall of the Gram-negative bacteria. Overall, the CE-samples showed for both bacteria types a stronger antibacterial effect than the PO samples due to the higher photodegradation efficiency [86]. Under UV irradiation, all ZnO-containing samples killed bacteria at an efficiency of 100%. Since the viability of *E. coli* and *S. aureus* is not affected by UV irradiation alone (Fig. S11), the decrease of viable *E. coli and S. aureus* on ZnO/PDMS membranes strongly indicates the effect of ROS generation from ZnO.

In general, there can be three mechanisms involved in the antibacterial activity related to ZnO NPs [87–89]. First, direct contact of bacteria with ZnO NPs can result in the disruption of the bacterial cell wall due to electrostatic interactions of the positively-charged ZnO nanoparticles (zeta potential of + 24 mV [90]) with the negatively-charged cell wall [91]. Second, the dissolution of Zn^2+^ ions into the solution is toxic to bacteria and zinc ions are known for their antimicrobial effect like other metal ions (e.g. silver, copper) [92, 93]. And third, the generation of ROS leads to oxidative stress by the oxidation of bacterial lipids, proteins and DNA. We measured Zn^2+^ concentrations in PBS of 0.2 ± 0.1, 4.4 ± 0.3 (= 0.068 mM) and 4.7 ± 0.3 mg/L (= 0.072 mM) for PDMS, PO_high and CE_high, respectively. While the control PDMS sample was affected by contaminated zinc, the detected Zn^2+^ concentrations for PO_high and CE_high were below the known minimum inhibitory concentration (MIC) of Zn^2+^ against *E. coli* and *S. aureus* (13.5 mM for ZnCl_2_) [94]. Hence, ROS generation by UV irradiation showed the strongest effect on the killing of bacteria as shown in Fig. 5.

The viability of *E. coli* in dark condition was ~ 5% for graphene oxide immobilized on ZnO tubes containing ~ 23 at.% zinc [95] and ~ 12% for membranes functionalized with graphene [96]. This antibacterial property was attributed to the release of zinc ions. For example, the antiviral activity of cotton membranes functionalized with ZnO containing 20 at.% zinc reached 99.9%, corresponding a Zn^2+^ concentration of 100 mg/L in acidic media before washing [97]. The amount of released Zn^2+^ in PBS from CE_high was 4.7 mg/L in phosphate buffer saline (PBS), as measured by ICP-MS. Without the leaching of zinc, a lower inhibition (~ 5%) of HCoV-OC43 mRNA may be obtained [98]. The antimicrobial activity is typically enhanced using photo-irradiation, reaching 100%, for materials containing zinc oxide or graphene [95, 96] or our membranes containing ZnO. Therefore, the ZnO/PDMS membranes provided a high antimicrobial activity in dark condition and under UV irradiation although they contained a relatively low metal content. The antibacterial recyclability of twist yarn containing 4% ZnO was investigated by measuring the concentration of Zn^2+^. The antibacterial activity of the twist yarn was stable after 20 times of washing [99]. In our work, we studied the recyclability of the ZnO/PDMS fibrous membranes for degrading rhodamine B. The decolorization of rhodamine B by PO_high under UV irradiation was maintained after five cycles (Fig. S10), which indicates their reusability. However, the release of zinc is strongly dependent on the media used [84].

PDMS is widely used as a material for applications with skin contact [100, 101] as well as in cell culture devices [102]. Although the ROS generated by ZnO could be toxic to human skin cells when in contact with the cells, ZnO NPs cannot penetrate through the stratum corneum to the dermis layer to inflict lasting damage [103]. Moreover, our membrane is meant to be sandwiched between two standard meshes of poly(vinylidene fluoride) or polypropylene. Therefore, the generated ROS on the membrane is not expected to harm the human skin.

The antiviral activity was not studied in our work due to the complexity of the test. Virus particles are reportedly to be more susceptible to Zn^2+^ and ROS than bacteria. In the dark, Zn^2+^ can penetrate into virus particles and inhibit DNA or RNA replication [104]. Moreover, ROS are known to damage the capsids of viruses and also induce crosslinking of capsid proteins, hereby preventing the binding of viruses on a host surface [105]. Both Zn^2+^ leaching and ROS generation were already studied in our work.

### Particle Filtration and Air Permeability Analysis

**Fig. 6:**
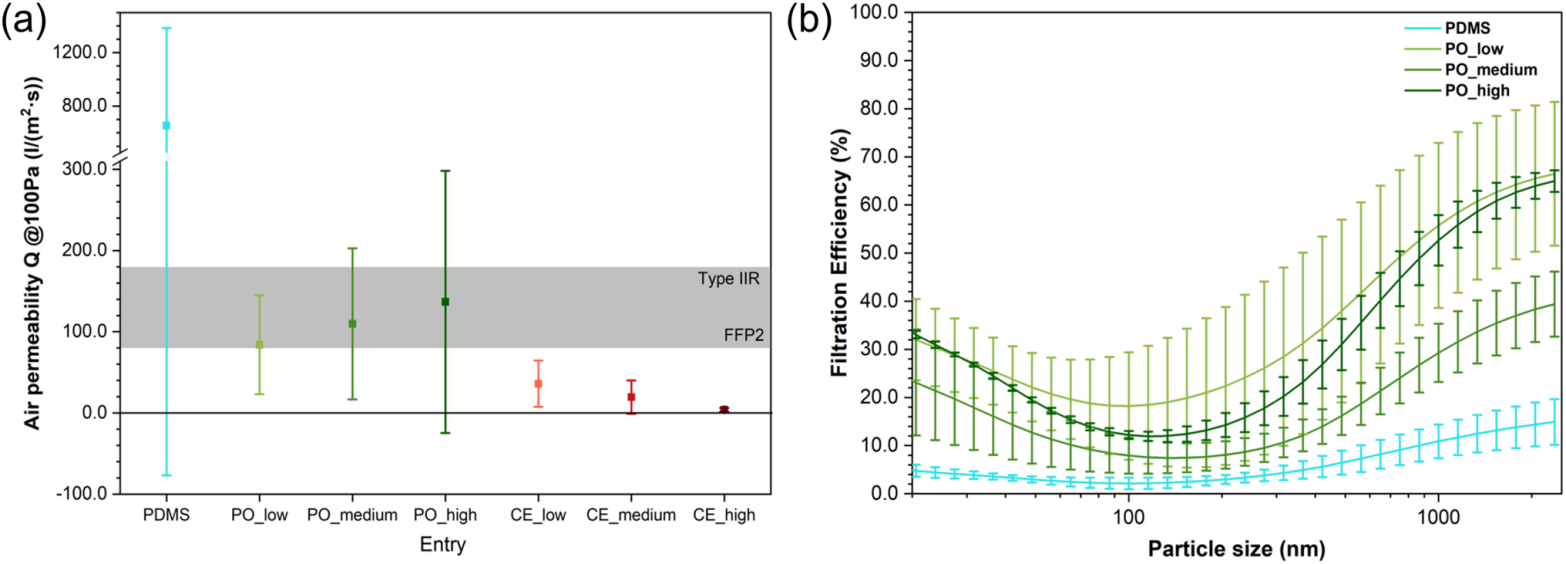
Air permeability **a** of PDMS fibers and ZnO/PDMS fiber membranes prepared by post-functionalization (PO) and colloidal electrospinning (CE). The gray-shaded area shows an acceptable range of airflow Q at 100 Pa. Particle filtration efficiency **b** of PDMS fibers and ZnO/PDMS fiber membranes prepared by PO

A simple irradiation-based sterilization function can be a significant property for membranes in applications such as wound covers or face masks. To investigate their potential application in air filtration, the filtration efficiency and air permeability properties of the functionalized membranes were characterized. Depending on the specific norm, aerosol-filtering membranes possess air permeability values in a relatively narrow range as to not cause breathing difficulties, while also not being too porous for aerosol droplets to pass unhindered. An acceptable range of airflow Q at 100 Pa between 80 and 180 L/m^2^ s was determined by testing examples of standardized medical and FFP2 masks, which was applied as the selection criterion for the filtration efficiency testing (Fig. 6a). PDMS-membranes showed the largest variation in air permeability, with only half of the samples found in the target region, despite keeping spinning parameters (solutions, voltage, time, distance) constant. Fluctuations in temperature and humidity during electrospinning are likely the cause of this discrepancy. Interestingly, the CE caused all samples to form air-impermeable films, as demonstrated by a negligible air permeability close to zero (Fig. 6a). When comparing the morphology of PO samples (Fig. 2a–c) and CE samples (Fig. 3d–f), the PO samples had larger pore size than CE samples. Moreover, it is possible that the ZnO NPs interfered with the thermal crosslinking procedure by physical mechanisms, for example as Pickering emulsifiers causing (partial) blending of the PDMS and PVP phases, or chemically by oxidation catalysis, as well as forming sterically inaccessible coordinative complexes with the silicone components. In contrast, the PO treatment resulted in the majority of PDMS samples barely changing their air permeability properties, as can be explained by a uniform coating of fibers. However, PO_high showed the highest variability in air permeability between the PO samples, showing that a too-high concentration of ZnO might just block the pores of the membrane.

Filtration mechanisms depend on several factors, where diffusion and electrostatic forces generally dominate below around 300 nm and physical hindrance outweighing above [106]. For the prepared PO and CE samples, the electrostatic charges should be mostly dissipated as a consequence of the alcohol washing, which is an established technique in filter analysis.

Aerosol filters often exhibit a minimum filtration efficiency at the overlap between diffusion (small) and physical size exclusion (large), known as the most penetrating particle size (MPPS). The MPPS for our membranes is found at relatively low sizes (approx. 100 nm, usually 100–300 nm), suggesting a stronger size-exclusion effect and a reduced diffusion mechanism. Generally, the filtration efficiency (FE) is not measured with particles in the MPPS range. The ZnO/PDMS fibrous membranes lacked electrostatic surface charges, which are the major contributor to the diffusion mechanism. Indeed, the membranes were prepared on an electrically conductive substrate and were not assembled by post-processing to form commercial masks. Hence, only the interception of larger particles around 1000 nm remains largely unaffected. Nevertheless, filtration efficiency characterization revealed a significant improvement of all PO samples compared to the original PDMS membranes (Fig. 6b). The filtration efficiency was calculated by Eq. (2). As noticeable from the SEM images, fibers partially fused and flattened during the treatment, which narrows and deforms the penetration path to cause higher filtration efficiency. Additionally, a significantly higher amount of smaller particles is also retained, which could be attributed to electrical isolation or regeneration of the charge by the ZnO particles in comparison to pure PDMS. While the alcohol washing certainly diminished the filtration capabilities of the membranes, these results demonstrate the applicability of this methodology with minor adjustments, such as a change in washing medium, sheath material or charge restoration.

Moreover, the thickness of the tested fibrous membranes was around 20 μm, which is much lower than the thickness of typical N95 filter layers (200–400 μm) [107]. The FE can be improved to meet the N95 requirement of 95% FE for particles with 100 nm diameter by increasing the thickness of the membrane during spinning or layering membranes to increase the thickness in post-processing. However, other norms such as those concerning medical (EN 14683: 95–98% at a particle diameter of 650–3000 nm) or community masks (SNR30000: 70% at a particle diameter of 1000 nm) specify much lower thresholds, which PO_low could be projected to reach even as they were (see Fig. 6b).

## Conclusions

Self-cleaning antibacterial ZnO/PDMS membranes were fabricated by two different processes. The polymer shell and PDMS core were electrospun with a coaxial nozzle and in-situ cured. The PVP shell was removed and the PDMS fibers were subsequently post-functionalized with ZnO under UV irradiation. In another approach, the ZnO-polymer shell and PDMS core were also electrospun with a coaxial needle and in-situ cured. The obtained fibers were then irradiated with UV followed by PVP removal. ZnO clusters were detected on PDMS by post-functionalization whereas homogeneously distributed ZnO NPs on PDMS was obtained by colloidal electrospinning. ZnO/PDMS fiber membranes prepared with post-functionalization could degrade a model dye and kill bacteria, hence indicating their potential for self-cleaning and antimicrobial properties. The ZnO/PDMS fibers prepared by post-functionalization could also filter particles effectively. The post-functionalization method could in principle be used to graft photoactive materials on PDMS for air filtration, oil/water separation and anti-biofouling.

Hypothetical cost calculations are difficult to project without translation into pilot-scale manufacturing. The coaxial electrospinning approach is likely more expensive than approaches based on current electrospun or melt-blown filters. However, wear time can be increased indefinitely until filter damage occurs due to handling/use. Especially in hospital settings where sterilization is of high concern but disruption from changing personal protective equipment (PPE) must be limited, this concept would also offer a crucial improvement, which could then be used to offset the increased cost. The assembly of filtration membranes in a commercial mask is an engineering-focused process that is beyond the scope of this study.

## Supplementary Information

Below is the link to the electronic supplementary material.

Supplementary file1 (DOCX 557 kb)

## Data Availability

The datasets generated and analyzed for this study are available from the corresponding authors on reasonable request.
