# Supplementary material for: Functional Fiber Membranes with Antibacterial Properties for Face Masks
Source: Adv Fiber Mater. 2023 May 17:1–15. Online ahead of print. doi: 10.1007/s42765-023-00291-7 (PMC10189208; doi:10.1007/s42765-023-00291-7)
Supplement: Supplementary file 1 — Supplementary file1 (DOCX 557 kb) [file 42765_2023_291_MOESM1_ESM.docx]

Supporting Information

Functional fiber membranes with antibacterial properties for face masks

Papada Natsathaporn,^a^ Gordon Herwig,^b^ Stefanie Altenried,^c^ Qun Ren,^c^ René M. Rossi,^b^ Daniel Crespy,^a,*^ and Fabian Itel^b,*^

^a^ Department of Materials Science and Engineering, School of Molecular Science and Engineering, Vidyasirimedhi Institute of Science and Technology (VISTEC), Rayong 21210, Thailand.

^b^ Empa, Swiss Federal Laboratories for Materials Science and Technology, Laboratory for Biomimetic Membranes and Textiles, Lerchenfeldstrasse 5, 9014 St. Gallen, Switzerland.

^c^ Empa, Swiss Federal Laboratories for Materials Science and Technology, Laboratory for Biointerfaces, Lerchenfeldstrasse 5, 9014 St. Gallen, Switzerland.

**Electrospinning of PDMS/PVA core-shell fibers**

The PDMS core solution was freshly prepared before electrospinning by mixing 0.4 g of Sylgard 184 elastomer and 0.04 g of curing agent (9:1 w/w ratio). 1.5 g of poly(vinyl alcohol) powder (PVA, Mowiol 18-88, M_w_ ~130,000 Da, Sigma-Aldrich) was dissolved in 8.5 g of DI water for use as polymer shell solution. The solutions were taken with a 1 mL syringe (inner diameter of 4.78 mm) and pumped with syringe pumps with flow rate of 0.1 mL∙h^-1^ for inner and 0.4 mL∙h^-1^ for outer channels of the blunt-end coaxial spinneret, respectively. The electrospinning was performed for 2 h with a positive high voltage of 7 kV at the needle, a needle to collector distance of 15 cm, a grounded heating plate covered with aluminum foil and with a set temperature of 100 ℃, a humidity between 40 – 50% and a room temperature between 25 and 35 ℃. After spinning, the aluminum foil containing the fibrous PDMS/PVA membranes was removed from the hot collector and further cured in an oven for 24 h at 100 ℃ to crosslink the PDMS core.

**Optimization of ZnO content of ZnO/PDMS fibers prepared by post-functionalization**

ZnO NPs were dispersed in IPA at different concentrations, specifically 0.035, 0.054, 0.108 0.168, 0.350, 0.540, 1.08 and 1.68 mg/mL. 1.68 mL of the dispersions were then transferred to a square well (3 x 3 cm) containing square (2 x 2 cm) PDMS fiber membrane on PLA mesh. The immersed PDMS fibers were exposed to UV irradiation (5 mW/cm^2^) for 2 h. The irradiated fiber membranes were rinsed with 2 mL of EtOH and dried with a stream of air. The obtained ZnO/PDMS fibers have theoretical ZnO content of 1.4, 2.1, 4.3, 6.7, 14.1, 22 ,43 and 67 wt%, respectively.

**Recyclability of ZnO/PDMS fibrous membranes for degrading rhodamine B**

100 µL droplets of rhodamine B solution (5 ppm in water) were deposited on PDMS, PO_high and CE_low membranes. Then, the samples were exposed to UV-irradiation (5 mW/cm^2^) for 1 h. Photographs of the fibrous membranes were taken with a camera (ISO 400, a shutter speed of 1/1000 and white balance of 5000 K). The fibrous membranes were then rinsed with 5 mL of EtOH to remove any residual dye and let dry completely, before the aforementioned process was repeated for 5 cycles.

**Influence of UV-irradiation on bacteria viability**

*E. coli* and *S. aureus* suspension in PBS were prepared as mentioned in the main manuscript to obtain the diluted concentration of bacteria of 3.09x10^4^ CFU/mL for *E. coli* and 2.02x10^5^ CFU/mL for *S. aureus*. 25 µL of the bacteria suspensions were pipetted into the wells of a 96-well plate and exposed to UV-irradiation (365 nm, 5 mW/cm^2^) for 15 min while the well plate was cooled on ice. The bacteria suspensions were then transferred to microtubes and the wells were rinsed again with 3 x 25 µL of PBS and transferred to the microtubes. The collected bacteria suspensions were spread on agar plates and incubated at 37 °C for 18 h. Bacteria colonies on agar plates were counted by an automatic colony counter (Scan 300, Interscience).

**Quantification of Zn^2+^ leaching from the PDMS and ZnO/PDMS membranes**

PDMS, PO_high and CE_high membranes were incubated in 250 µL of phosphate-buffered saline (PBS) for 2 h at 30 °C. The solution was filtered through 10 kDa filters (Amicon Ultra-0.5 Centrifugal Filter Unit, Millipore) to remove any possible ZnO nanoparticles and other solids that could interfere with the measurement. The samples were diluted 100-times using ultrapure nitric acid solution (2% HNO_3_, Normatom®, VWR chemicals) in 15 mL Falcon tubes prior to analysis using an inductive couple plasma mass spectrometer (ICP-MS, Agilent 7900). Zn^2+^ concentrations were validated with known zinc nitrate concentrations in nitric acid solutions.

**
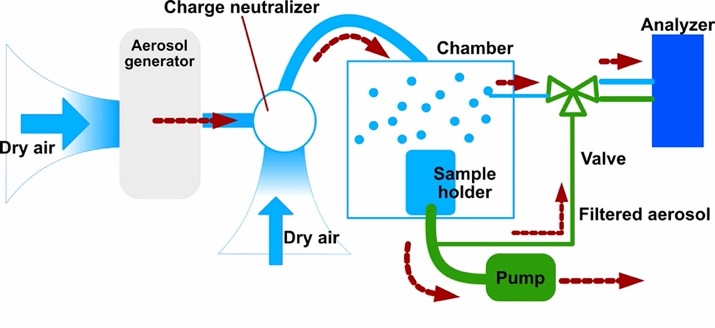
**Fig. S1 Scheme depicting the particle filtration testing. The dark red arrows show the flow of aerosol.


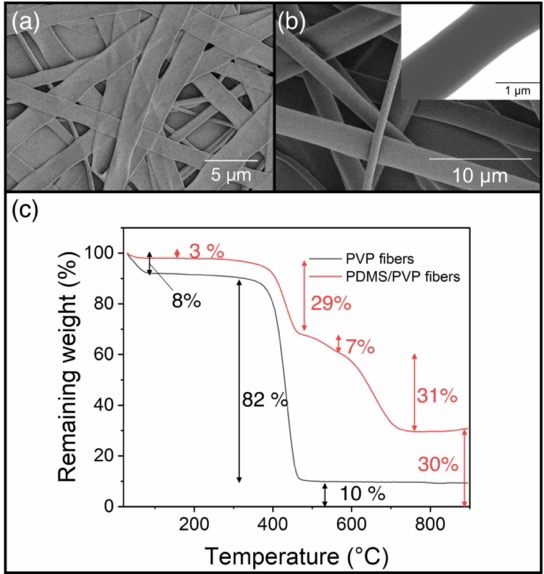
Fig. S2 Analysis of control samples. SEM micrographs (a-b) of flat-ribbon PVA/PDMS fibers (a) and PDMS/PVP fibers (b) with inset of TEM image presenting the core-shell structure. Thermogravimetric analysis (TGA) traces (c) of PVP fibers and PDMS/PVP fibers.


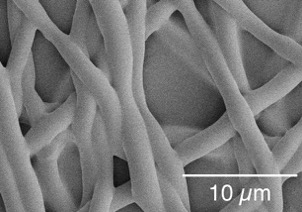
Fig. S3 Analysis of control samples. SEM micrographs of bare PDMS fibers after PVP removal.


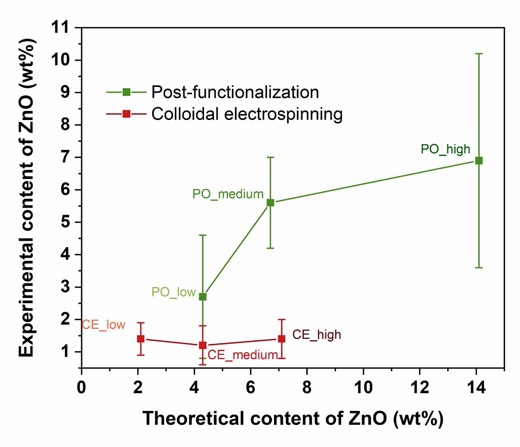
Fig. S4 Correlation plot between theoretical content of ZnO on PDMS and experimental content of ZnO measured by EDX.


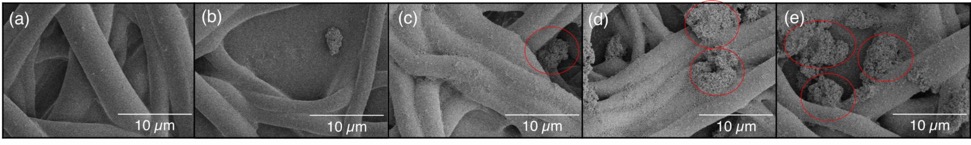
Fig. S5 SEM micrographs of ZnO/PDMS post-functionalized fibers with different ZnO loadings: (a) 1.4 wt%, (b) 2.1 wt%, (c) 22 wt%, (d) 43 wt% and (e) 67 wt%. Red circles show aggregates of ZnO NPs on fibers.


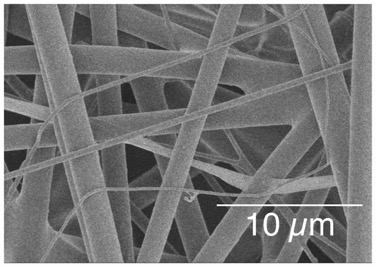


Fig. S6 SEM micrographs of ZnO/PDMS fibers prepared by colloidal electrospinning (CE) before PVP removal with 10.7 wt% ZnO.


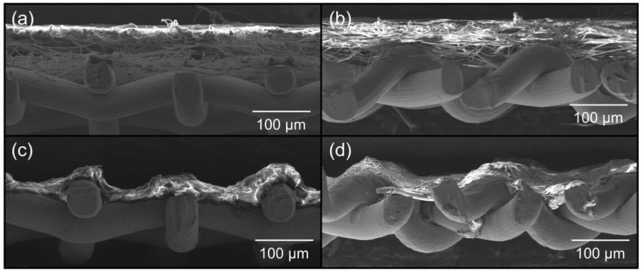
Fig. S7 SEM cross-section images of ZnO/PDMS fibrous membranes before (a,b) and after (c,d) funcionalization and shell removal of PO_high (a,c) and CE_low (b,d).

Table S1 Average tensile strength and displacement of PDMS and selected ZnO/PDMS fibrous membranes

| **Entry** |  | **Thickness**  **(µm)** |  | **Tensile Strength (MPa)** |  | **Displacement (mm)** |
| --- | --- | --- | --- | --- | --- | --- |
| PDMS fibers |  | 18 ± 6 |  | 1.7 ± 0.3 |  | 56.2 ± 4.7 |
| PO_high |  | 36 ± 12 |  | 0.7 ± 0.2 |  | 49.7 ± 3.0 |
| CE_low |  | 41 ± 16 |  | 0.8 ± 0.3 |  | 16.1 ± 1.1 |


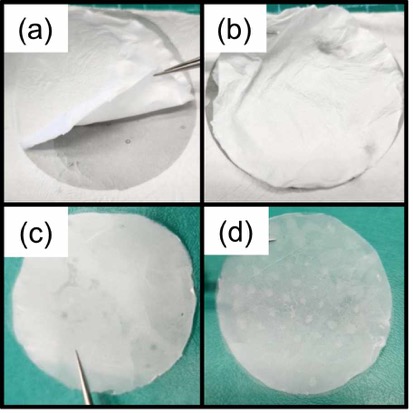
Fig. S8 Photographs of ZnO/PDMS fibroud membranes before (a,b) and after (c,d) funcionalization and shell removal of PO_high (a,c) and CE_low (b,d).

**
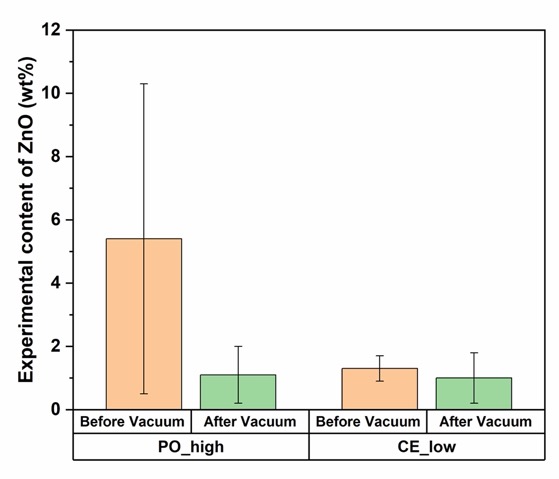
**Fig. S9 Average ZnO content on PO_high and CE_low before and after the application of vacuum.


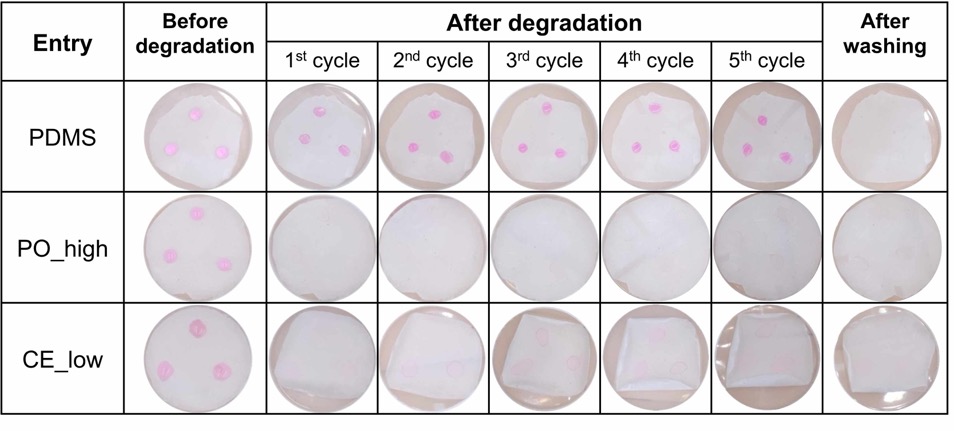
Fig. S10 Photographs of PDMS and ZnO/PDMS fibrous membranes (PO_high and CE_low) before and after 1 h of photodegradation tested for 5 cycles, and after washing with ethanol.


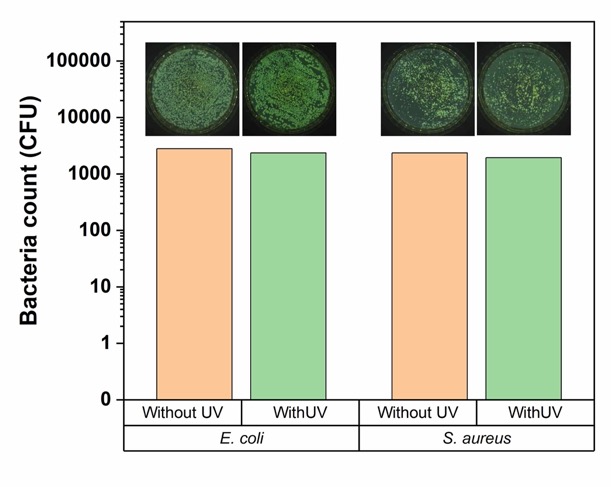
 Fig. S11 Effect of UV-irradiation on bacterial viability. Bar graph and photographs of viable bacteria (CFU), Gram-positive *Staphylococcus aureus* (*S. aureus*) and Gram-negative *Escherichia coli* (*E. coli*), without and with UV-irradiation for 15 min.
